# Obinutuzumab in Rituximab-resistant and recurrent membranous nephropathy: a case-series

**DOI:** 10.1007/s40620-025-02224-6

**Published:** 2025-02-20

**Authors:** Krita Sridharan, Basu Gopal, Scott Wilson, Alan Pham, Holly Hutton

**Affiliations:** 1 Department of Nephrology, St Vincent’s Hospital, Melbourne, VIC Australia; 2 Department of Nephrology, The Alfred Hospital, Melbourne, VIC Australia; 3 Monash University Central Clinical School, Melbourne, VIC Australia; 4 Melbourne, VIC Australia; 5 Department of Anatomical Pathology, The Alfred Hospital, Melbourne, VIC Australia

**Keywords:** Membranous nephropathy, Treatment-resistant, Obinutuzumab

## Abstract

**Background:**

Membranous nephropathy (MN) is a common cause of nephrotic syndrome in adults, with high risk of progression to end-stage kidney disease when untreated. Rituximab is commonly used in its treatment however many patients do not respond. Obinutuzumab is a novel anti-CD20 monoclonal antibody for which there is increasing observational evidence in treatment-resistant membranous nephropathy. The majority of evidence for its use relates to anti-phospholipase A2 receptor-(PLA2R) associated membranous nephropathy.

**Methods:**

This was a single-centre case-series of all patients at a tertiary nephrology centre in Melbourne, Australia, treated with Obinutuzumab for membranous nephropathy, between January 2023 and June 2024. All patients who received Obinutuzumab were included in this case-series, irrespective of PLA2R status.

**Results:**

Out of 5 patients with treatment-resistant membranous nephropathy, 3 had PLA2R-associated membranous nephropathy which had previously been refractory to, or relapsed on Rituximab therapy. All 3 patients with PLA2R-positive membranous nephropathy achieved complete immunological and clinical remission after receiving Obinutuzumab. The case of secondary PLA2R-negative membranous nephropathy only achieved partial remission after Obinutuzumab before unexpectedly dying from another cause. The case of recurrent PLA2R-associated membranous nephropathy in a renal allograft did not respond to Obinutuzumab.

**Conclusion:**

This case-series supports the existing evidence in favour of Obinutuzumab for treatment-resistant PLA2R-associated membranous nephropathy. To our knowledge it is the first reported use of Obinutuzumab in sarcoidosis-associated membranous nephropathy.

**Graphical abstract:**

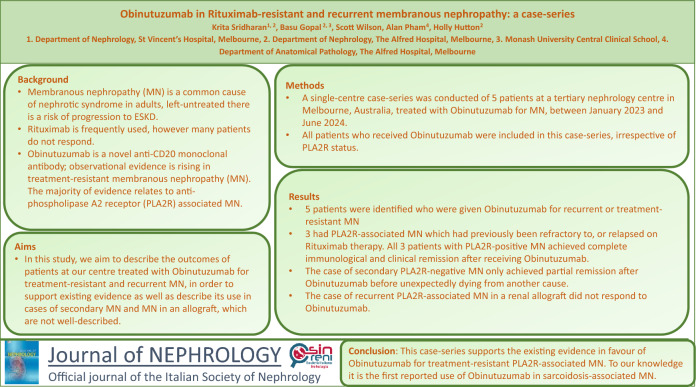

## Introduction

Membranous nephropathy (MN) is a common cause of nephrotic syndrome in adults [1]. Untreated, up to 50% of patients with membranous nephropathy may develop end-stage kidney disease (ESKD) within ten years [1, 2]. The recognition of membranous nephropathy as an autoimmune, antibody-mediated condition, has led to the use of B-cell-depleting agents in its treatment [1]. Rituximab, a chimeric anti-CD20 monoclonal antibody, is commonly used in the treatment of membranous nephropathy, with several trials published including GEMRITUX, STARMEN, MENTOR and RI-CYCLO [3–6]. However up to 40% of patients do not respond to Rituximab, and its use is associated with recurrent disease [6].

Obinutuzumab is a humanised type II anti-CD20 monoclonal antibody, with superior B-cell cytotoxicity to Rituximab in in vitro studies [7]. It can cause rapid and prolonged peripheral CD19 + B-cell depletion, as demonstrated in the NOBILITY trial [8]. This is related to a distinct mode of binding to the CD20 antigen, with greater affinity for the Fc receptor on effector cells, leading to greater antibody-dependent cellular cytotoxicity to B cells than type I monoclonal antibodies such as Rituximab [9].

There is emerging evidence for the efficacy of Obinutuzumab in treating Rituximab-resistant membranous nephropathy, with several case series now published, primarily in PLA2R-positive membranous nephropathy.[10–13] A recent case-series has been published of Obinutuzumab as first-line treatment in untreated primary membranous nephropathy, with promising results [14]. We present a single-centre experience of the use of Obinutuzumab in treatment-resistant or recurrent membranous nephropathy, with 5 cases reported: 3 cases of PLA2R-associated membranous nephropathy, 1 case of secondary membranous nephropathy (sarcoidosis), and 1 case of recurrent primary membranous nephropathy in a renal allograft.

## Methods

The study period was from January 2023 to September 2024. It was a single-centre retrospective study at a large tertiary metropolitan nephrology unit. Patients had received Obinutuzumab for either recurrent or treatment-resistant membranous nephropathy. Treatment-resistant membranous nephropathy was defined as ongoing nephrotic syndrome with or without positive PLA2R despite previous Rituximab ± other immunosuppression. Recurrent membranous nephropathy was defined as recurrence of nephrotic syndrome despite previous immunosuppressive therapies. Patients were included irrespective of PLA2R status, and patients with secondary causes of membranous nephropathy, as well as renal transplant recipients were included. All patients were followed up during routine outpatient appointments and their data were collected from hospital electronic medical records.

Data collected included sex, age, comorbidities, and previous therapies. Laboratory data included urine protein creatinine ratio (uPCR), albumin creatinine ratio (uACR) where uPCR was not reported, serum creatinine (sCr) and albumin, serum PLA2R, and CD19 B-cell counts. Other data included histopathology and secondary screening results (imaging, infection serology, pulmonary function testing and others). All patients received a standard dose of Obinutuzumab 100 mg test dose on day 1, 900 mg on day 2, and 1000 mg on day 15.

The outcomes were clinical and immunological remission. Complete remission was defined as urine PCR of < 30 mg/mmol, partial remission was defined as urine PCR > 30 mg/mmol and < 300 mg/mmol with normalisation of serum albumin. Immunological remission was defined as normalisation of PLA2R titre. All patients gave informed consent to inclusion in this case-series.

## Results

Of 5 cases reported, 3 achieved complete clinical and immunological remission after Obinutuzumab, all of whom have maintained remission through to the end of the study period. All 3 patients had PLA2R-associated membranous nephropathy. One case of secondary membranous nephropathy associated with sarcoidosis only attained partial clinical remission before dying unexpectedly from another cause. One case of refractory membranous nephropathy in a renal allograft did not respond to Obinutuzumab; this patient was tissue-PLA2R-positive but serum PLA2R-negative. All patients had full CD19+ suppression after Obinutuzumab. Time to complete remission ranged from 1 to 9 months (Table 1).

**Table 1:** Results pre and post-Obinutuzumab

	Case 1	Case 2	Case 3	Case 4	Case 5
Serum albumin at diagnosis/recurrence (g/L)	21	29	32	18	31
PCR at presentation (mg/mmol)	459	1400	438	1393	421
sCr at presentation (mcmol/L)	52	133	60	86	143
Serum PLA2R at presentation (RU/mL)	1270	19	50	Negative	Negative
Kidney biopsy	Diffuse GBM thickening, subepithelial spikes and deposits. Staining for IgG, IgM, IgG4, C3, IgA, C1q, C4. No fibrosis	Diffuse GBM thickening, subepithelial spikes and deposits. Staining for IgG, IgM, IgG4, C3, C4, C1q, mild staining for IgA. Mild fibrosis	Diffuse GBM thickening, subepithelial spikes and deposits. Staining for IgG, IgM, C1q, C3, C4, mild staining for IgA. 10–15% fibrosis	Diffuse GBM thickening, staining for IgG, IgM, C3, IgG4, IgA, C1q. Minimal fibrosis	Diffuse GBM thickening, mesangial matrix expansion, 15–20% IFTA, PLA2R strong diffuse staining. C4d negative. on PTC, strongly positive on peripheral glomerular capillary loop walls
Previous immunomodulatory therapies	Rituximab, tacrolimus	Rituximab, modified Ponticelli	Rituximab, tacrolimus, prednisolone, cyclosporin, azathioprine, prednisolone	Prednisolone, rituximab, tacrolimus	Rituximab, tacrolimus, mycophenolate, prednisolone, IVIG
Serum albumin post Obinutuzumab (g/L)	41	44	40	39	31
PCR/ACR post Obinutuzumab (mg/mmol)	25 (PCR)	10 (ACR)	6.7 (ACR)	269 (PCR)	740 (PCR)
sCr post Obinutuzumab (mcmol/L)	88	158	74	134	199
Serum PLA2R post Obinutuzumab (RU/mL)	Undetectable	Undetectable	Undetectable	N/A	N/A
CD19 post Obinutuzumab	< 0.1%	< 0.1%	< 0.1%	< 0.1%	< 0.1%
Clinical response	Remission	Remission	Remission	Partial remission	No remission
Immunological response	Remission	Remission	Remission	N/A	N/A
Time to complete remission	6 months	9 months	1 month	N/A	N/A

*SCr* serum creatinine, *GBM* glomerular basement membrane, *Ig* immunoglobulin, *PCR* protein-creatinine ratio, *ACR* albumin creatinine ratio, *IFTA* interstitial fibrosis and tubular atrophy, *PTC* peritubular capillaries, *IVIG* intravenous immunoglobulin

### Case 1—PLA2R-associated membranous nephropathy

A 72-year-old woman presented in July 2022 with a 4-month history of nephrotic syndrome with preserved renal function, uPCR 458 mg/mmol, and serum albumin 21 g/L. There were no relevant comorbidities. Serum PLA2R antibody was positive with titre 1270 RU/mL. Investigations for secondary causes of membranous nephropathy demonstrated positive hepatitis B core antibody, with negative surface antigen, and were otherwise negative. Kidney biopsy confirmed membranous nephropathy, with no fibrosis.

Antiproteinuric therapy was initiated. Nevertheless, proteinuria worsened, with uPCR 1134 mg/mmol at one-month follow-up. Due to doubling of proteinuria and high initial PLA2R, Rituximab was given, 1 g two weeks apart. She was covered with entecavir for hepatitis B reactivation prophylaxis.

**Fig. 1:**
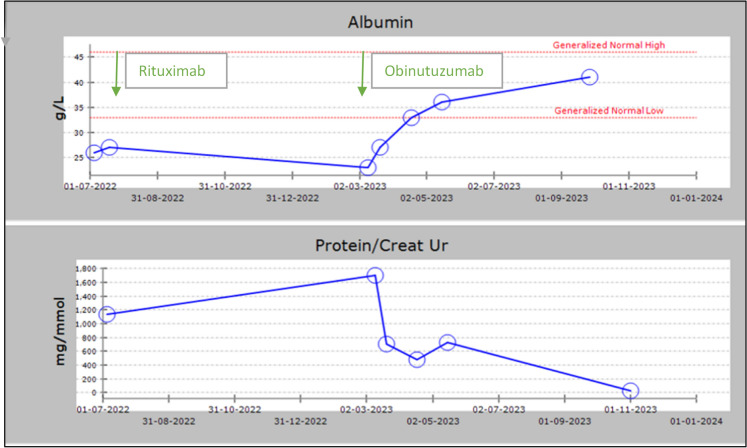
Graph of trend in serum albumin and urine protein-creatinine ratio, Case 1. Graph of serum albumin (g/L) and urine protein-creatinine ratio (mg/mmol) for Case 1. Studies in July 2022 demonstrated heavy proteinuria and hypoalbuminaemia. Subsequently, the patient received treatment with Rituximab. Repeat investigations in February 2023 demonstrated worsening proteinuria and hypoalbuminaemia. Subsequent treatment with Obinutuzumab. Significant increase in serum albumin and reduction in urine protein-creatinine ratio with most recent studies demonstrating complete remission

Six months after Rituximab, PLA2R titre was still elevated at 111 RU/mL and heavy proteinuria persisted with uPCR 1700 mg/mmol and serum albumin 23 g/L. Tacrolimus was commenced, with a trough target of 5–7 ng/mL, and a decision was made to proceed to Obinutuzumab in March 2023. Four months after Obinutuzumab administration serological remission was attained with negative serum PLA2R and full CD19 suppression (< 0.1%), with improvement in uPCR to 483 mg/mmol and serum albumin to 36 g/L (Fig. 1). Complete clinical remission was attained by month-6 post Obinutuzumab with uPCR 25 mg/mmol. At latest follow-up, 13 months post Obinutuzumab, there is ongoing complete remission, with uPCR 22 mg/mmol and serum albumin 41 g/L, despite B-cell reconstitution (Fig. 1).

Tacrolimus is being weaned to cessation given the excellent response to Obinutuzumab.

### Case 2—PLA2R-associated membranous nephropathy

A 74-year-old man presented with recurrent primary membranous nephropathy in September 2023. He was diagnosed with primary membranous nephropathy in 2017, after presenting with nephrotic syndrome with uPCR 803 mg/mmol, serum albumin 26 g/L and renal impairment with sCr 129 mmol/L, with positive serum PLA2R.

His relevant past medical history included hypertension and hyperlipidaemia, and he had no secondary cause for membranous nephropathy.

He was initially treated with renin–angiotensin–aldosterone (RAAS) blockade and modified Ponticelli regimen (cyclical corticosteroids and cyclophosphamide), after which he achieved partial remission.

He relapsed with nephrotic syndrome in May 2019 with uPCR 1400 mg/mmol, serum albumin 19 g/L and PLA2R-positive at 41 RU/mL. He was then treated with Rituximab, 1 g two weeks apart, and he attained complete immunological remission and partial clinical remission. From 2020 to 2022 he had ongoing sub-nephrotic proteinuria, with undetectable PLA2R, and stable kidney function.

**Fig. 2:**
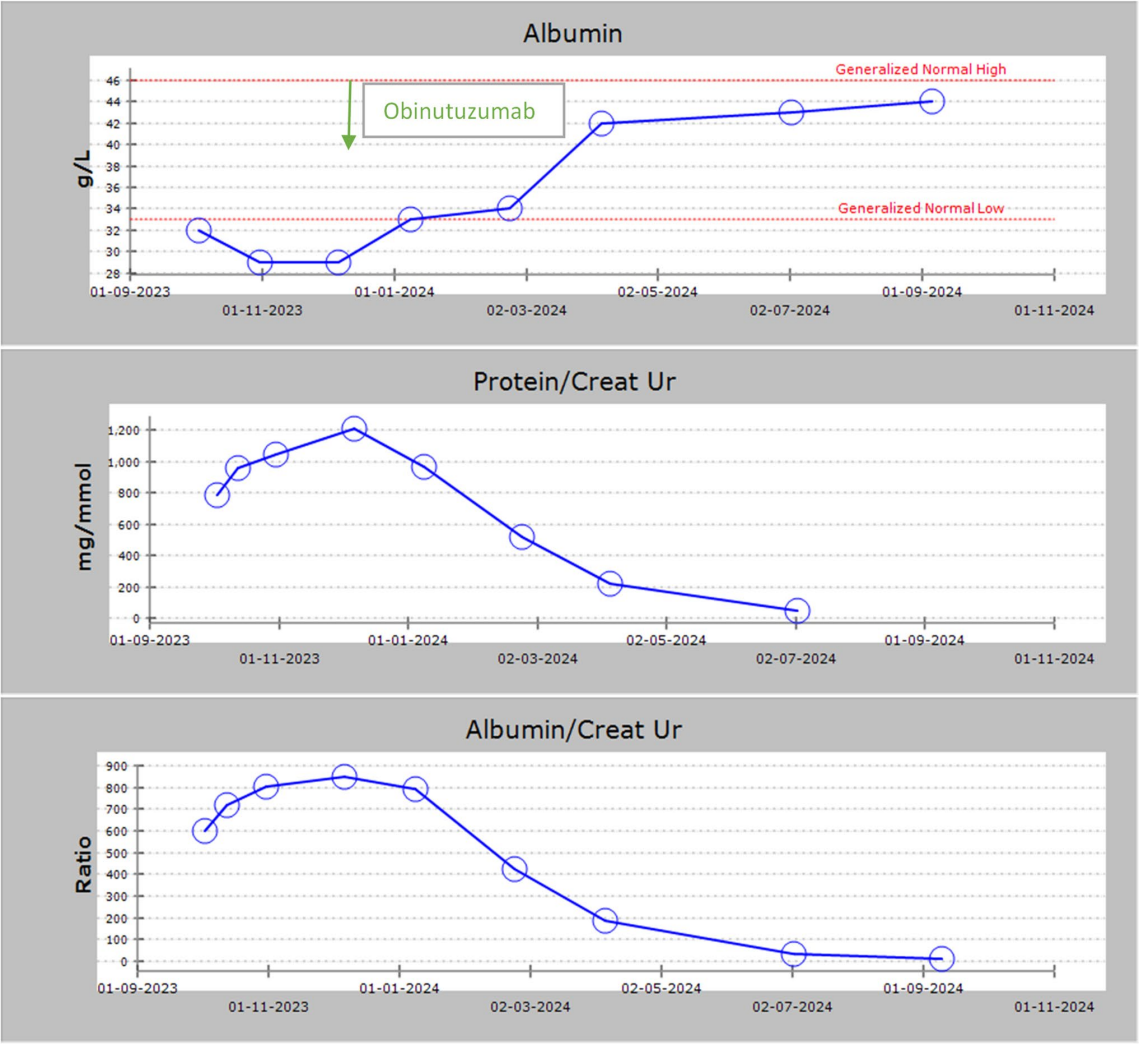
Graph of trend in serum albumin and urine albumin/protein-creatinine ratio, Case 2. Graph of serum albumin (g/L) and urine protein-creatinine ratio and albumin-creatinine ratio (mg/mmol) for Case 2. Studies in September, November and December 2023 demonstrated heavy proteinuria and hypoalbuminaemia. Subsequently the patient received treatment with Obinutuzumab. Repeat investigations in January 2024 demonstrated gradually improving serum albumin and proteinuria with normalisation of serum albumin despite ongoing nephrotic range protenuria. Complete remission of proteinuria (uPCR < 30mg/mmol) was achieved by month 9 after Obinutuzumab

His nephrotic syndrome relapsed in September 2023 with uPCR 800 mg/mmol and serum albumin 29 g/L with positive PLA2R titre 19 RU/mL. Repeat kidney biopsy demonstrated features of membranous nephropathy with mild fibrosis. He was given Obinutuzumab in December 2023. In February 2024 he had normalisation of PLA2R titre and serum albumin, improvement in uPCR to 518 mg/mmol and suppression of B cells (CD19 < 0.1%). As of his most recent follow up, in September 2024, he remains in immunological remission and is, for the first time since initial diagnosis, in full clinical remission with urine ACR 10 mg/mmol and serum albumin 44 g/L (Fig. 2).

### Case 3—PLA2R-associated membranous nephropathy

A 43-year-old woman was seen in our clinic with frequently relapsing primary membranous nephropathy. She had initially been diagnosed in 2002 after presenting with nephrotic syndrome with proteinuria 13 g/day with preserved renal function. Renal biopsy confirmed primary membranous nephropathy with positive serum PLA2R. She had no relevant past medical history nor any secondary cause for membranous nephropathy.

She was treated with antiproteinuric therapy as well as prednisolone and azathioprine, achieving remission.

She subsequently went on to have multiple relapses, in 2003, 2012, and 2016; treated with azathioprine and prednisolone in 2003, then cyclosporin and prednisolone followed by partial remission. In June 2017 she had another nephrotic relapse, serum PLA2R 84 RU/mL; repeat kidney biopsy confirmed ongoing membranous nephropathy, with mild fibrosis. She was treated with Rituximab with partial remission. She had two further nephrotic relapses between 2018 and 2019, both treated with further Rituximab. After a relapse in 2021 she was commenced on tacrolimus with a target range of 4–6 ng/mL. She achieved partial clinical and full immunological remission with this, and improved to full clinical remission in 2023.

In March 2024, tacrolimus was weaned. Two months after cessation she relapsed with urine PCR 438 mg/mmol, serum albumin 32 g/L and PLA2R- positive at 50 RU/mL. Tacrolimus was restarted and Obinutuzumab was given. One month following Obinutuzumab, she attained complete remission with uACR 16 mg/mmol and negative serum PLA2R, and remains in complete remission 4 months after Obinutuzumab with a plan to wean tacrolimus.

### Case 4—secondary membranous nephropathy

A 42-year-old man was diagnosed with membranous nephropathy in July 2021 after presenting with nephrotic syndrome with preserved renal function. Urine PCR was 1393 mg/mmol, serum albumin 18 g/L. Serum PLA2R antibody was negative. Kidney biopsy confirmed membranous nephropathy, with minimal fibrosis. PLA2R staining on biopsy was not performed. Antiproteinuric therapy was initiated.

Relevant past medical history included Hodgkin’s lymphoma treated in 2000 with chemotherapy (doxorubicin, bleomycin, vinblastine and dacarbazine), now in remission.

Screening for secondary causes of membranous nephropathy revealed mediastinal and hilar lymphadenopathy and multiple lung nodules. Lymph node core biopsy demonstrated non-caseating granulomas, consistent with a diagnosis of sarcoidosis.He was commenced on high dose prednisolone for the sarcoidosis. Due to ongoing nephrotic syndrome, he was then given tacrolimus with a target trough level 6-8 ng/mL and Rituximab 1 g two weeks apart for the membranous nephropathy. Therapy was complicated by steroid-induced diabetes.

His membranous nephropathy partially responded to this therapy, with concurrent response of sarcoidosis.

Tacrolimus was discontinued in month 10 due to acute kidney injury (AKI), with peak creatinine 315 µmol/L resolving to a new baseline of 130–150 µmol/L. Due to increasing nephrotic range proteinuria following this, tacrolimus was recommenced with a lower trough target (3–6 ng/mL) and further Rituximab given. There was no response, despite B-cell suppression.

Given persistent proteinuria, in February 2023 a kidney biopsy was repeated, demonstrating ongoing active membranous nephropathy with 40% interstitial fibrosis and tubular atrophy. There was also evidence of pulmonary sarcoid recurrence. The prednisolone dose was increased for sarcoidosis treatment. B-cell reconstitution was noted, and the decision was made to trial Obinutuzumab for ongoing active membranous nephropathy, particularly due to poor tolerance of ongoing steroid therapy. The patient received Obinutuzumab in May 2023. At follow-up 9 months post, there was partial remission with uPCR reduced to 215 mg/mmol (see Table 1), sCr 145 µmol/L, and full CD19 suppression (< 0.1%).

Unfortunately, in March 2024, the patient died suddenly and unexpectedly at home. Post-mortem was not performed due to cultural reasons. Discussion at a multidisciplinary meeting revealed a consensus opinion of the likely cause of death to be an acute cardiac event, in view of significant cardiovascular risk factors. Cardiac sarcoid was thought to be unlikely in view of a negative PET scan.

### Case 5—recurrent primary membranous nephropathy in renal allograft

A patient was diagnosed with primary membranous nephropathy in 2009 at the age of 37 years, which was managed with the modified Ponticelli regimen. Serum PLA2R was negative, kidney biopsy PLA2R stain was not performed. He progressed to ESKD in 2014 and commenced peritoneal dialysis.

In 2016, he received a deceased donor (after brain death) kidney transplant and was maintained on conventional tacrolimus, prednisolone, and mycophenolate mofetil immunosuppression. Graft function was excellent in the early years post-transplant with no rejection episodes.

In May 2018, the patient developed rising proteinuria with uPCR 289 mg/mmol and new hypoalbuminaemia (serum albumin 27 g/L), with relatively preserved renal function. Transplant kidney biopsy demonstrated global thickening of the glomerular basement membrane (GBM) with diffuse IgG and IgG4 staining and weak granular IgM staining, consistent with recurrent membranous nephropathy. There was no evidence of rejection. Serum PLA2R antibody was negative, and staining for PLA2R on biopsy was not performed. Rituximab, 1 g, two weeks apart was given for recurrent membranous nephropathy, and antiproteinuric therapy was maximised. There was partial response with improvement to < 1 g/day proteinuria.

In 2021, the patient’s membranous nephropathy relapsed. uPCR was 420 mg/mmol and weak de novo donor-specific antibodies (DSAs) to two class II antigens were detected. Biopsy demonstrated advanced membranous nephropathy with sclerosis. Immuno-peroxidase stain for PLA2R showed strong diffuse GBM staining. C4d stain was negative on peritubular capillaries but strongly positive on peripheral glomerular capillary loop walls. Serum PLA2R remained negative. Further Rituximab was given, as well as intravenous immunoglobulin to offset DSA production and any contribution to the proteinuria from possible antibody-mediated rejection.

**Fig. 3:**
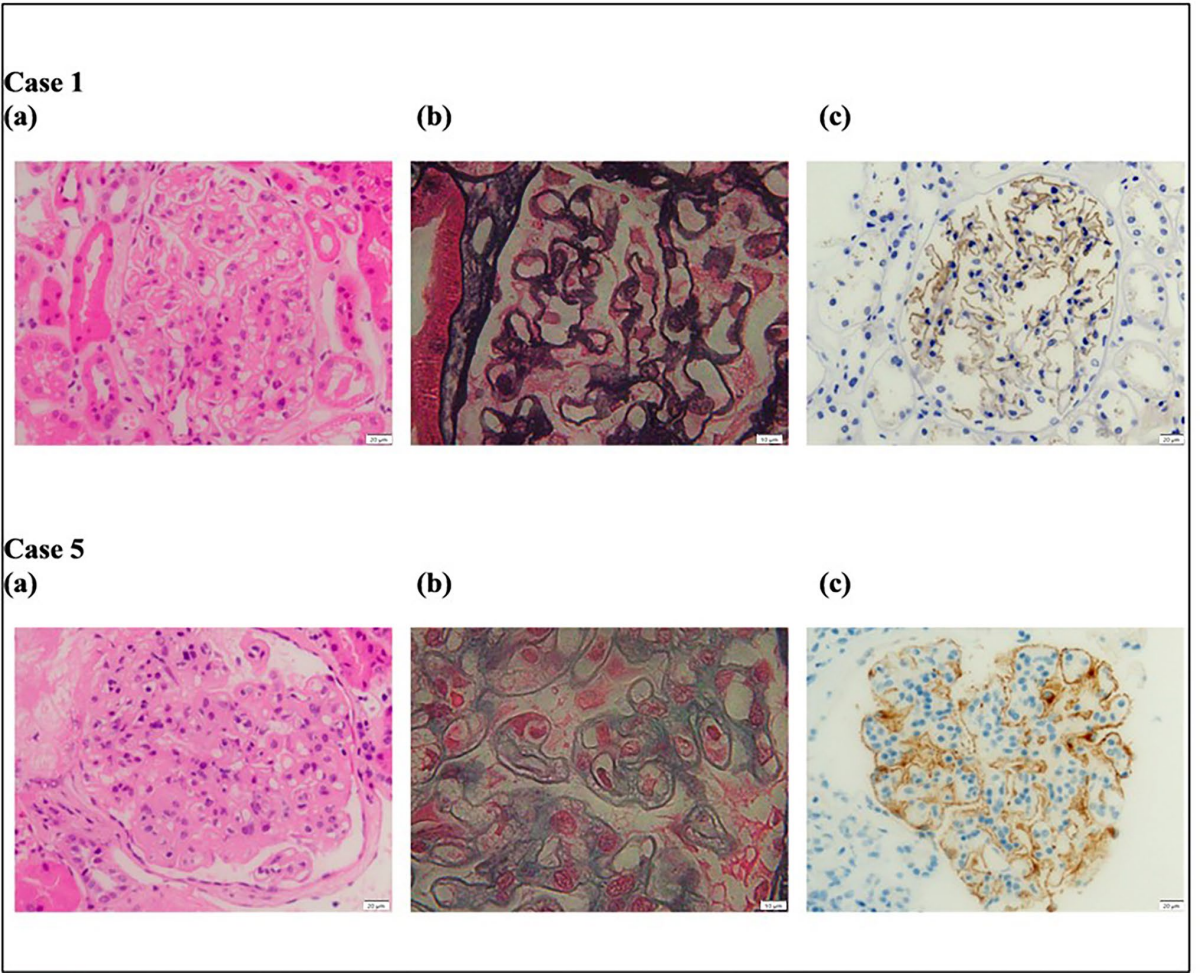
Kidney biopsy images, Case 1 and Case 5. Case 1(a): diffuse thickening of the glomerular basement membrane (H&E × 400), 9 Jun 2022. Case 1(b): glomerular basement membrane spiking with subepithelial deposits (periodic acid silver methenamine × 1000), 9 Jun 2022. Case 1(c): granular glomerular basement membrane immunoperoxidase staining with IgG, 9 June 2022. Case 5(a): diffuse thickening of the glomerular basement membrane (H&E × 400), 5 Apr 2023. Case 5(b): glomerular basement membrane encircling deposits with “split” appearance (periodic acid silver methenamine × 1000), 5 Apr 2023. Case 5(c): positive immunoperoxidase staining for PLA2R

Nephrotic range proteinuria persisted, with urine PCR > 300–400 mg/mmol, despite partial CD19 suppression (0.2%). Renal function worsened, with sCr 153 µmol/L in January 2023. Repeat biopsy one year following the second course of Rituximab demonstrated ongoing active primary membranous nephropathy with strong PLA2R staining in peripheral capillary loops, with 20% interstitial fibrosis and tubular atrophy (IFTA). C4d staining was again present in glomeruli but not in the peritubular capillaries (Fig. 3).

In view of ongoing membranous nephropathy with inadequate response to Rituximab, he proceeded to Obinutuzumab in May 2023.

As yet, there has been no response to Obinutuzumab. A follow-up biopsy 6 months post-Obinutuzumab demonstrated ongoing active membranous nephropathy with PLA2R staining of unchanged intensity and 20–25% IFTA. At his most recent follow up, 14 months post Obinutuzumab, there is ongoing proteinuria, with most recent uPCR of 466 mg/mmol, serum albumin of 31 g/L, despite full CD19 suppression. Renal function has deteriorated, with recent sCr in the range of 250–300 µmol/L.

The patient remains on transplant immunosuppression, as well as monthly intravenous immunoglobulin. He developed central serous chorioretinopathy from steroids, therefore his prednisolone dose has been weaned to 1 mg/day, with tacrolimus trough levels maintained between 7 and 9 ng/mL. Given his deteriorating renal function, he is being worked up for dialysis initiation.

## Discussion

We present 5 cases of treatment-resistant or recurrent membranous nephropathy. All cases of PLA2R-associated membranous nephropathy have demonstrated complete immunological and clinical response to Obinutuzumab, with time to complete remission ranging from 1 to 9 months. The patient with sarcoidosis-associated membranous nephropathy underwent partial remission, despite evidence of ongoing pulmonary sarcoid activity, however unfortunately she died unexpectedly due to a presumed cardiovascular event. The case of recurrent PLA2R-associated membranous nephropathy in a renal allograft has not demonstrated response to Obinutuzumab and his graft function continues to deteriorate. None of our patients reported adverse effects or infusion reactions attributable to Obinutuzumab.

The majority of published evidence for Obinutuzumab in refractory membranous nephropathy relates to primary membranous nephropathy. Sethi et al. reported a case series of ten patients with largely Rituximab-refractory membranous nephropathy (six of whom were PLA2R-positive on serum or biopsy, and four of whom had a transplant) [10]. Complete or partial remission was achieved by 85.7% of patients with Obinutuzumab, and all of these patients with available follow-up data at 12 months had maintained remission. A 2020 case series of three patients with Rituximab-refractory PLA2R-associated membranous nephropathy demonstrated complete immunological remission in all three patients followed by partial clinical remission in two [11]. Another article reported two cases of PLA2R-associated membranous nephropathy who both achieved immunological and clinical remission with Obinutuzumab after failure of Rituximab [12]. There has been a recent report of successful use of Obinutuzumab in the treatment of IgG-4-related membranous nephropathy [15]. A 2024 case-series of 18 patients with PLA2R-associated membranous nephropathy demonstrated either partial or complete remission in 94.4% of patients, 61.1% of whom had unsatisfactory response to Rituximab, with a median time to remission of 2.7 months [13].

The improved response to Obinutuzumab in Rituximab-refractory membranous nephropathy is thought to relate to more profound and sustained CD19+ B cell depletion compared to Rituximab. One theory regarding Rituximab-resistant disease in the setting of peripheral B cell depletion is that pathogenic B cells are hidden in sanctuary sites such as tissue or bone marrow. Obinutuzumab is likely more efficacious in depleting bone marrow and tissue-resident B cells compared with Rituximab, which may help explain its benefits [16].

Treatment of secondary membranous nephropathy is typically focused on treating the underlying cause. However, secondary membranous nephropathy is also thought to be an antibody-mediated illness, with documented association with thrombospondin type-1 domain-containing 7A and nerve epidermal growth factor-like 1 antibodies, and likely yet undiscovered antibodies [17]. As such, it may be postulated that antibody-depleting agents would also lead to improvement in secondary membranous nephropathy. In case 4, the patient’s sarcoidosis was difficult to treat, and steroid intolerance led us to consider other treatment options. The lack of complete response in this case may relate to active sarcoidosis as the primary pathology.

This case series adds to previous reports of efficacy of Obinutuzumab in refractory PLA2R-associated membranous nephropathy. It is also the first report, to our knowledge, of Obinutuzumab use for refractory sarcoidosis-associated secondary membranous nephropathy, and one of only few reports in an allograft. We report partial remission in the case of secondary sarcoidosis-associated membranous nephropathy, but no efficacy in the allograft. Obinutuzumab was well-tolerated in all patients. A phase III randomised controlled trial (RCT) is currently underway assessing the efficacy and safety of Obinutuzumab compared to tacrolimus in primary membranous nephropathy and is expected to be completed by 2028 (NCT04629248). Another phase III RCT looking at Obinutuzumab vs corticosteroid and cyclophosphamide in primary membranous nephropathy (REMIT) is planned to start soon.

This and previous case series show that Obinutuzumab is an effective, often rapid treatment in treatment-resistant and recurrent primary membranous nephropathy. At this stage, the role of Obinutuzumab for secondary or PLA2R-negative membranous nephropathy is unclear, but may be reasonable to consider in disease refractory to existing agents.

## Data Availability

Data sharing is not applicable to this article as no datasets were generated or analysed for this article.
